# Frequency of *GJB*2 mutations, *GJB*6‐D13S1830 and *GJB*6‐D13S1854 deletions among patients with non‐syndromic hearing loss from the central region of Iran

**DOI:** 10.1002/mgg3.780

**Published:** 2019-06-04

**Authors:** Hossein Naddafnia, Zahra Noormohammadi, Shiva Irani, Iman Salahshoorifar

**Affiliations:** ^1^ Department of Biology, Science and Research Branch Islamic Azad University Tehran Iran

**Keywords:** autosomal recessive nonsyndromic hearing loss, connexin 26, connexin 30, *GJB*2 mutation, *GJB*6 mutation

## Abstract

**Background:**

In the present study, we investigate the prevalence of the *GJB*2 gene mutations, and deletions in the *GJB*6 gene, namely del (*GJB*6‐D13S1830) and del (*GJB*6‐D13S1854), in patients with autosomal recessive non‐syndromic hearing loss (ARNSHL) from the central region of Iran.

**Methods:**

One hundred and thirty‐one unrelated ARNSHL cases from the central part of Iran were recruited. Among them, 81% (106 cases) had at least two affected relatives. Coding and noncoding regions of the *GJB*2 gene were sequenced. Multiplex PCR was used for analysis of del (*GJB*6‐D13S1830) and del (*GJB*6‐D13S1854) deletions in *GJB*6.

**Results:**

The *GJB*2 variants were found in 16.79% (22/131) of the patients. The pathogenic variants were 21/131 (16.03%). The nonpathogenic variants were 1/131 (0. 07%). Allele frequency of the c.35delG as the pathogenic variant was the most common with 59.52% (25/42). The remaining pathogenic variants were c.235delC, p.T8M, p.R32H, p.R143Q, p.R143W, c‐23+1G>A. The only nonpathogenic variant was polymorphism p.V27I. Further segregation analysis showed that variant of p.R143Q might have incomplete penetrance. None of the patients had targeted deletions in the *GJB*6 gene.

**Conclusion:**

In comparison with reports from other areas of Iran, c.35delG demonstrates the highest frequency within the central region (accounting for 57.14% of cases), probably resulting from the founder effect and consanguineous marriage. The pathology of ARNSHL in such patients could be attributed to defects in Connexin 26 encoded by *GJB*2.

## 1 INTRODUCTION

As one of the most common sensory disorders worldwide, hearing loss (HL) is a heterogeneous group of disorders in which both genetic and environmental risk factors are contributing to the disease etiology (Hashemzadeh, Farhud, & Patton, 2007). At least 50% of prelingual hearing loss in industrialized countries has been referred to genetic causes (Marres, 1998). The incidence rate of cases with prelingual congenital hearing loss is 1 per 1,000 live births in the general population (Petit, Levilliers, & Hardelin, 2001). At least one out of 500 newborns has permanent bilateral sensorineural hearing impairment of more than 40 dB (Marres, 1998). Before 5 years old age, the prevalence increases to 2.7 per 1,000. Also, some degrees of deafness affect the natural relationship in 4% of people under 45 and 10% of people over 65 years of age and older (Davis, 1989; Marres, 1998; Petit, 1996; Petit et al., 2001).

Nonsyndromic hearing loss (NSHL) is the most frequent form of neurosensory deafness, accounting for almost 70% of inherited hearing impairments (Schrijver, 2004). The majority of NSHL cases have autosomal recessive pattern of inheritance (Schrijver, 2004). To date, over 100 genes have been reported for NSHL (http://hereditaryhearingloss.org/). Mutation in the *GJB*2 (MIM: 121011), *GJB*3 (MIM: 603324), *GJB*6 (MIM: 604418) genes has been detected for the majority of inherited cases of NSHL.

Mutation in the *GJB*2 is the major cause of autosomal recessive nonsyndromic hearing loss (ARNSHL) in several populations (del Castillo & del Castillo, 2011; Chan & Chang, 2014). The *GJB*2 gene which is located on the DFNB1 locus (13q11‐q12), codes for the connexin 26 protein. This protein presents in the human cochlea from the 22nd week of embryonic development. It recycles potassium ions within the inner ear, particularly in the cells of the limbus, the spiral ligament, and the Corti organ (Alexandrino et al., 2009; Forge et al., 2003).

Over 200 pathogenic mutations in the *GJB*2 have already been reported (Lerer et al., 2001). Most of them are responsible for recessive NSHL, whereas others account for dominant forms, either NSHL or HL in relation with dermatological disorders (syndromic HL, Stenson et al., 2009). The c.35delG has been detected as the most frequent *GJB*2 pathogenic mutation in Mediterranean, North American and European patients with NSHL (Petersen & Willems, 2006; Stenson et al., 2009) This mutation creates a premature stop codon which is resulted from deletion of a guanine residue within a stretch of Gs between nucleotide positions 30 and 35 (Denoyelle et al., 1997). *GJB*2 is coexpressed with *GJB*6 in different cells of the cochlea. *GJB*6 deletions have been reported in association with HL homozygously or in compound heterozygous state with a *GJB*2 mutation (Lerer et al., 2001, del Castillo et al., 2005, 2002).

Genetic analysis of *GJB*2 and *GJB*6 has been widely conducted in different parts of Iran, but there is no published report for the Qom province. Hence, we aimed to investigate these genes in patients with congenital ARNSHL in the central region of Iran, Qom province.

## 2 METHODS

### 2.1 Ethical compliance

This study was approved by the Ethical committee of Islamic Azad University, Science and Research Branch, Tehran (approval No. 33805). Informed consent was obtained from patients before commencement of the research.

### 2.2 Subjects

In the present study, hearing impaired individuals were obtained from who referred to Pouya Genetic counseling center and Qom Welfare Organization during the period between August 2013 and November 2016 were studied.

The clinical status of the subjects was verified by an Otolaryngologist. The criteria for inclusion of the subjects were cases with NSHL greater than 40 dB. Individuals with known deafness‐related syndromes and HL resulted from acquired environmental factors like infections, trauma or ototoxic drugs were excluded from the analysis.

Finally, 131 patients were selected according to the test conditions. The age of patients varied between 3 and 80 years old. Sixty‐five out of the 131 patients (49.6%) were females and 66 (50.4%) were males. Among the 131 probands, 19% (25/131) were sporadic cases of HL (17 of which with parent's consanguinity), and 81% (106/131) had at least two affected relatives with bilateral HL (familial cases, 87 of which with parent's consanguinity). DNA extraction and amplification analyses were performed.

### 2.3 DNA analysis

Genomic DNA was extracted from EDTA anticoagulated whole blood using the Puregene Blood Core Kit (Cat. No. 8510400, Qiagen, Germany) according to the manufacturer's instruction. In order to determine mutations of the *GJB*2, exons 1 and 2 including the flanking intronic and UTR regions had been amplified through PCR (The Veriti^®^ Thermal Cycler, Applied Biosystems, USA) and sequenced by Sanger sequencing (ABIPrism@ 3130XL Genetic Analyzers, ThermoScientific, USA) as previously described by Frei et al.(2002). The primers for complete length of exon 2 and noncoding exon 1 were chosen from Frei et al. (2002) and Kelsell et al. (1997).

All the patients were also screened for deletions in *GJB*6 (D13S1830, D13S1854) and the coding exon of *GJB*6.

Deletion breakpoint junctions of the *GJB6* gene were amplified by using the multiplex PCR technique with three pairs of primers, as described previously by del Castillo et al. (2005, 2002).

The PCR products were visualized on 1.2% agarose gel and extracted by gel extraction kit (Gel Extraction Kit, Qiagen). PCR products were bidirectionally sequenced using same primers. DNA sequences were aligned with reference sequences of *GJB*2 (NM_004004.5) and *GJB*6 (NM_001110219.2) genes.

## 3 RESULTS

The mutation c.35delG was the most prevalent pathogenic variant with 61.9% allele frequency, of which 57.1% of the mutant alleles were in homozygous state and 4.8% were compound heterozygous (Table 1). Genotype‐phenotype correlation analysis showed that all cases with a c.35delG variation had severe to profound HL with similar degrees in both ears.

**Table 1:** *GJB*2 gene mutations observed in the present study

Genotypes	No. probands/total sample	No. probands/probands with alterations in *GJB*2
c.35delG/c.35delG	12/131 = 9.16%	12/22 = 54.55%
c.235delC/c.235delC	2/131 = 1.52%	2/22 = 9%
c.23C>T/c.23C>T (p.T8M)	1/131 = 0.76%	1/22 = 4.55%
c.‐23+1G>A/c.‐23+1G>A	1/131 = 0.76%	1/22 = 4.55%
c.‐23+1G>A/c.35delG	1/131 = 0.76%	1/22 = 4.55%
c.428G>A/c.428G>A (p.R143Q)	1/131 = 0.76%	1/22 = 4.55%
c.427C>T/c.427C>T (p.R143W)	1/131 = 0.76%	1/22 = 4.55%
c.95G>A/c.95G>A (p.R32H)	2/131 = 1.52%	2/22 = 9%
Total (pathogenic)	21/131 = 16%	21/22 = 95.5%
c.79G>A/c.79G>A (p.V27I)	1/131 = 0.76%	1/22 = 4.55%
Total (nonpathogenic)	1/131 = 0.76%	1/22 = 4.55%
Total	22/131 = 16.8%	22/22 = 100%

**Figure 1:**
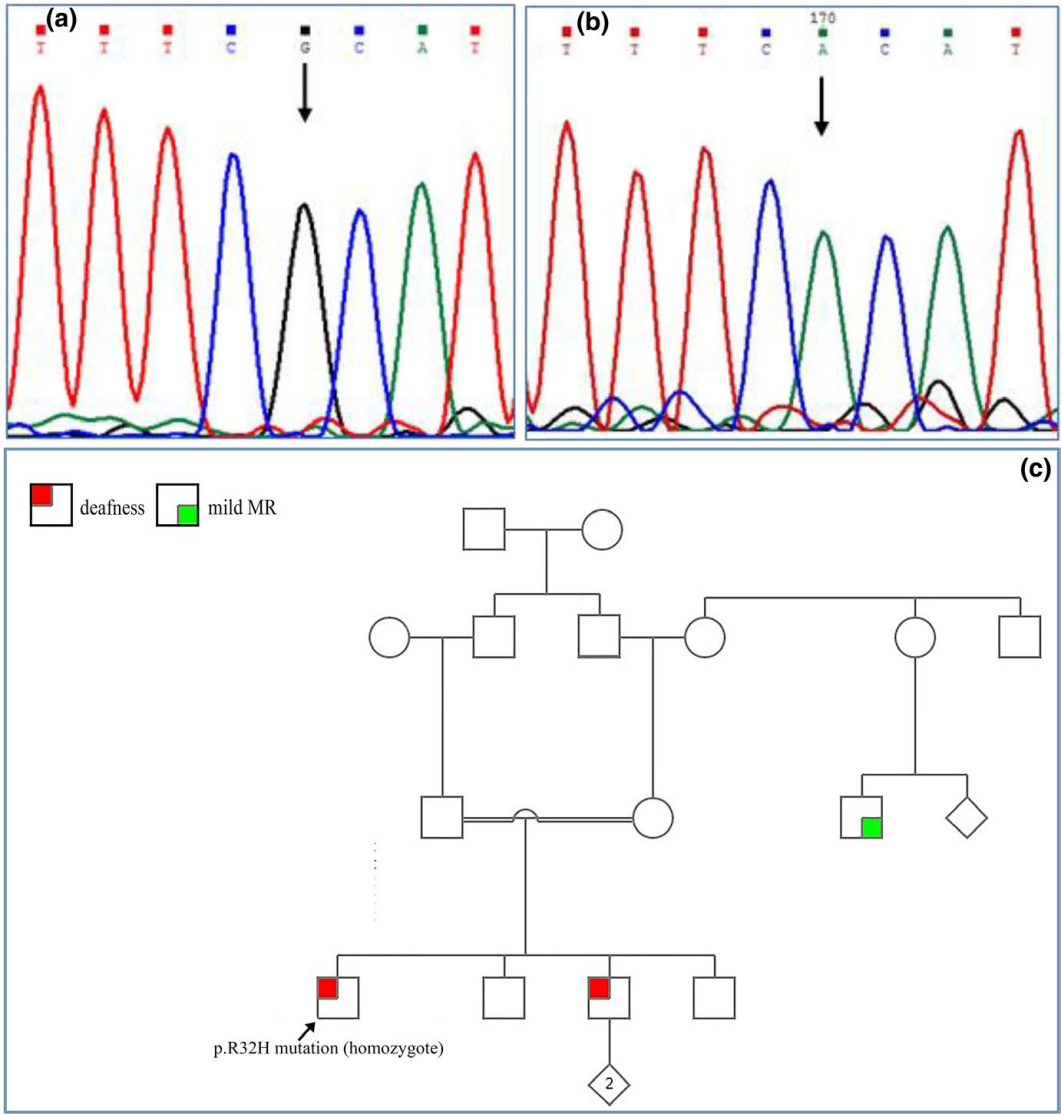
Sequence of the R32H (c.95G>A) mutation. a: wild‐type, b: homozygote mutant, c: The pedigree of this mutation

**Figure 2:**
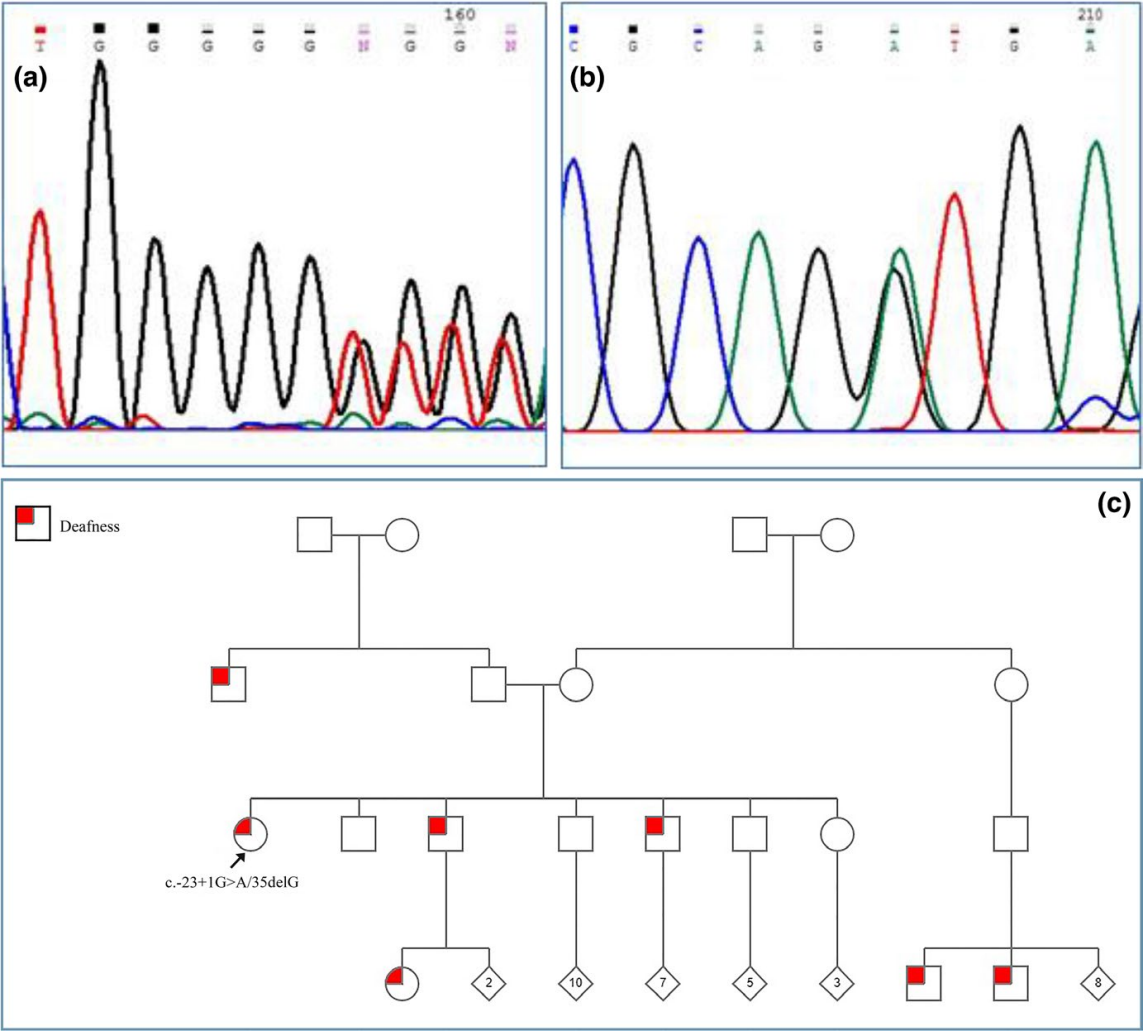
Sequences of the compound heterozygote (c.‐23+1G>A/35delG). a: The heterozygote sequence of G deletion at position 35; b: The heterozygote sequence of G to A transition at the splice site position; c: The pedigree of this mutation

We found two patients (9.5%) with the p.R32H mutation which substitutes Arginine residue with Histidine at codon 32 (Figure 1). The c.235delC mutation was detected in two other subjects (9.5%). All of the c.23C>T, c.428G>A and c.427C>T mutations (homozygote) were shown in one of the patients (4.8%) in the present study. A splice site mutation c.‐23+1G>A in the *GJB*2 gene was detected in two patients, one (4.8%) being homozygote, and the other (4.8%) showing compound heterozygosity (Figure 2). The p.V27I variant (had been previously considered as a benign polymorphism) was detected in one patient. None of the deaf patients carried the *GJB*6 mutation.

According to the findings, the mutations p.R32H (four alleles), c.235delC (four alleles), c.‐23+1G>A, c.23C>T (two alleles), p.R134W (two alleles), p.R134Q (two alleles) and p.V27I (one allele) are the most frequent mutations after c.35delG in the *GJB*2 gene in the patients from central regions of Iran, respectively.

Our study showed that the c.35delG mutation was found in 9.92% of 131 unrelated cases of ARNSHL, either sporadic or familial. Thus this mutation is consistent with 59.5% (25/42) of the pathogenic alleles detected (Table 2).

**Table 2:** Allele frequencies of pathogenic variants

Allele type	Total (% of all mutated alleles)	Mutation type
c.35delG	25/42 = 59.52%	Frame shift
c.235delC	4/42 = 9.5%	Frame shift
c.23C>T (p.T8M)	2/42 = 4.7%	Missense
c.‐23+1G>A	3/42 = 7.1%	Splice site
c.428G>A (p.R143Q)	2/42 = 4.7%	Missense
c.427C>T (p.R143W)	2/42 = 4.7%	Missense
c.95G>A (p.R32H)	4/42 = 9.5%	Missense

One hundred and four cases of the patients (79%) were siblings of consanguineous marriages. Out of the 22 pathogenic and nonpathogenic variations identified in the *GJB*2 gene, 18 were the result of consanguineous marriage (Table 3).

**Table 3:** The distribution of mutant alleles in analyzed patients with ARNSHL

Genotype	Sporadic cases (25)		Familial cases (106)		Total
	Parental consanguinity		Parental consanguinity		
	(17) +	(8) −	(87) +	(19) −	131
c.35delG/c.35delG	1		7	4	12
c.235delC/c.235delC	1		1		2
c.23C>T/c.23C>T			1		1
c.‐23+1G>A/c.‐23+1G>A			1		1
c.‐23+1G>A/c.35delG			1		1
p.R143Q/p.R143Q			1		1
p.R143W/p.R143W			1		1
p.R32H/p.R32H			2		2
p.V27I/p.V27I	1				1

Abbreviation: ARNSHL, autosomal recessive non‐syndromic hearing loss.

## 4 DISCUSSION

Hearing loss is an etiologically heterogeneous trait. Mutation in the *GJB*2 gene is the most common cause of ARNSHL worldwide and has been detected in many ethnic populations (del Castillo et al., 2002; Morell et al., 1998; Park, Hahn, Chun, Park, & Kim, 2000). In addition, deletions that truncate the *GJB*6 gene but also remove a regulatory element needed for the expression of *GJB*6 causes ARNSHL. In the present study, we investigated the prevalence of *GJB*2 and *GJB*6 mutations in cases of familial and sporadic ARNSHL and determined the frequency of mutations in the population of the central part of Iran.

**Figure 3:**
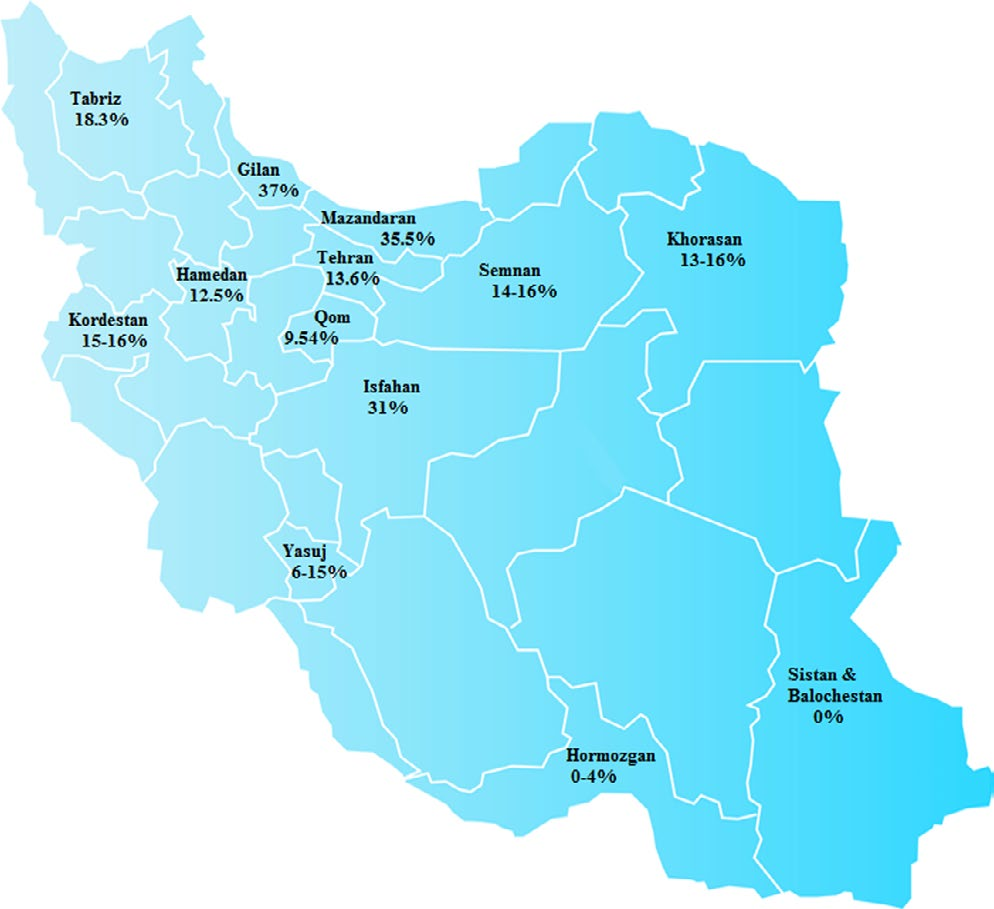
The frequency of the deletion c.35del G in the different regions of IRAN

Based on the measured allele frequency of *GJB*2 c.35delG mutation (9.54%) in the central region of Iran (Qom province), this variation is the most common mutation among cases of autosomal recessive nonsyndromic bilateral moderate to profound sensorineural hearing loss. Thus, the deletion c.35delG represents a hot spot mutation. This result is in agreement with other reports. Several studies have shown that the frequency of the c.35delG mutation in *GJB*2 varied from 28% to 63% among individuals with ARNSHL, and from 10% to 30%, among sporadic cases (Bonyadi, Esmaeili, Abhari, & Lotfi, 2009; Esmaeili, Bonyadi, & Nejadkazem, 2007; Feldmann et al., 2004; Gasparini et al., 2008). In a study reported in Mazandaran province (Gualandi et al., 2002), the most common reported mutation was c.35delG (35.5%). Another study (Samanich et al., 2007) reported the rate of homozygous c.35delG mutation to be 31% in Isfahan province while it was 12.5% in Hamadan province (Najm, Khosh, Pourfatemi, & Kahrizi, 2004). According to another study, the frequency of the c.35delG mutation was 13.6% and 18.3% in Tehran and Tabriz, respectively (Rezaei, Broojeni, & Movahedi, 2010). Figure 3 shows the frequency of the deletion c.35del G in the different regions of IRAN.

The second most common mutation in the *GJB*2 gene in this study was c.235delC and p.R32H, each composing 9.5% of the pathogenic alleles. The remaining ARNSHL associated mutations had only one representative within our study sample. The V27I has been reported in the Brazilian population and is also common in the East Asian countries. This missense mutation has previously been introduced as a polymorphism without pathological effects and unrelated to HL (Hashemzadeh et al., 2005; Shafeghati, Ebrahimi, Mohseni, OstadiF, & Poujafari, 2006). The p.V27I was found in one of our patients. The splice site mutation c.‐23+1G>A is positioned in the intron donor splice site and is anticipated to disrupt the splicing pattern, which results in no detectable mRNA (Castro et al., 2013). In a study on Iranian Azeri‐Turkish patients (Abe, Usami, Shinkawa, Kelley, & Kimberling, 2000), 16 (1 homozygous and 15 heterozygous) families out of the 174 families had c.‐23+1G>A mutation, with all of the 16 families demonstrating consanguineous marriage. The present study showed that this mutation was detected in two patients (one homozygote and one compound heterozygote with 35delG), both having parental consanguinity.

The p.R143Q mutation segregates with dominantly inherited hearing loss, which ramps from normal or mild to moderate or severe (Kudo et al., 2000). Bonyadi, Fotouhi, and Esmaeili (2011) reported the p.R143Q mutation as compound heterozygous with c.35delG in an Iranian Azeri‐Turkish patient affected with profound hearing loss. The proband had two affected sisters with same mutations and a normal sister who carried heterozygous p.R143Q mutation, showing this variant had partial penetrance. Huang et al. (2013) reported a dominant R143Q mutation in *GJB*2 in three patients from two unrelated families among 9,041 patients from unrelated families in mainland China. In our study, the p.R143Q was identified in one patient. Further genetic analysis of the family showed that two other sibs (a sister and a brother) had profound hearing loss and both being homozygous for the p.R143Q. Parents were healthy heterozygote carriers (with consanguineous marriage); also the affected sister had a heterozygote healthy son. In this family, according to the pedigree, the inheritance pattern of the p.R143Q mutation is autosomal recessive. But in previous studies, this mutation has been described as autosomal dominant (Kudo et al., 2000), indicating the incomplete penetrance of this mutation. The observed incomplete penetrance of p.R143Q in this family could be due to some other possible modifier genes cosegregating with the mutation which remains to be determined. Because of the incomplete penetrance of this mutation, it is difficult to exactly predict the precise recurrent risk for the carriers, and severity of HL can vary from normal to profound. Based on our findings, the high frequency of c.35delG than other pathogenic variants the *GJB*2 gene among the ARNSHL may be due to the founder effect and high proportion of consanguineous marriages. Lack of mutation in *GJB*6 among Iranian population may reinforces the founder's hypothesis.

## 5 CONCLUSION

According to this study and other studies conducted in all parts of Iran the contribution of the *GJB*2 gene pathogenic variants in ARNSLH was the highest among all of the other genes. The discovery of mutations in *GJB*2 gene as one of the main factor in the genetic occurrence of a HL has a significant effects on early diagnosis in the general population. An important topic about HL genetic counseling is that the severity of HL due to *GJB*2 gene is extremely variable and unpredictable, even among the patient members of a family, so providing a panel of common *GJB*2 gene mutations along with common mutations in other genes related HL helps in better genetic counselling, prevention and care.

## CONFLICT OF INTEREST

The authors have no conflict of interest.

## AUTHOR CONTRIBUTION

ZN was involved in conceptualization of the project. HN, ZN, SI, and IS were involved in project design and preparation of the manuscript. HN was involved in data collection and lab work. HN and ZN were involved in analyses of data and statistics. All authors read and approved the manuscript.

## COMPLIANCE WITH ETHICAL STANDARDS

### Informed consent

Informed consent was obtained from all individual participants included in the study.
